# Mercury Spikes Indicate a Volcanic Trigger for the Late Ordovician Mass Extinction Event: An Example from a Deep Shelf of the Peri-Baltic Region

**DOI:** 10.1038/s41598-019-39333-9

**Published:** 2019-02-28

**Authors:** Justyna Smolarek-Lach, Leszek Marynowski, Wiesław Trela, Paul B. Wignall

**Affiliations:** 1 Faculty of Earth Sciences, University of Silesia, Sosnowiec, 41-200 Poland; 2 Polish Geological Institute ‒ National Research Institute, Kielce, 25-953 Poland; 3 School of Earth and Environment, University of Leeds, Leeds, LS2 9JT UK

## Abstract

The Late Ordovician mass extinction (LOME) was the second largest Phanerozoic crisis, but its cause remains elusive. Several triggering mechanisms have been proposed over the years, including bioevolutionary events, oceanographic changes, and geotectonic processes. Here, we report the presence of Hg spikes in the Zbrza PIG-1 borehole from the Upper Ordovician deep shelf sections of the peri-Baltic region. A strong positive anomaly in the lower late Katian (Hg/TOC = 2537.3 ppb/wt%) was noted. No correlation between Hg and TOC (R^2^ = 0.07) was distinguished in the Hirnantian, although several positive anomalies were found. Because the Hg/Mo ratio showed trends very similar to those of Hg/TOC, it seems likely that TOC values reflect the redox conditions. In order to evaluate the role of anoxia in levels of Hg enrichment several redox indicators were measured. These showed that the elevated mercury values in the Hirnantian are not caused by anoxia/euxinia because euxinic biomarkers (maleimides and aryl isoprenoids) are present in very low abundance and pyrite framboids are absent. In total, positive Hg/TOC anomalies occur in the lower late Katian, at the Katian - Hirnantian boundary, and in the late Hirnantian. The lack of a strong Hg/TOC correlation, Ni enrichments, and the absence of ‘anoxic indicators’ (no biomarkers, no framboids, low Mo concentration) at these levels, supports the interpretation that Hg enrichment is due to enhanced environmental loading. We conclude that our Hg and Hg/TOC values were associated with volcanic pulses which triggered the massive environmental changes resulting in the Late Ordovician mass extinction.

## Introduction

The Late Ordovician mass extinction (LOME), the second largest of the Phanerozoic^1^, occurred in two pulses^2,3^, an initial early Hirnantian phase (in the *extraordinarius-ojsuensis* graptolite zones at ~445 Ma), associated with a major glacioeustatic sea-level fall^4^ and a late Hirnantian phase (in the *persculptus* zone at ~444 Ma), which coincided with climate warming, post-glacial marine transgression, and anoxia^5,6^. The causes of LOME remain the subject of extensive debate. Several triggering mechanisms have been proposed over the years^7^, including bioevolutionary events^8,9^, oceanographic changes^5,10–12^, and geotectonic processes (e.g. a possible mid-Ordovician superplume)^13,14^.

As has been shown recently, continental arc volcanism has a significant impact on climate change on a global scale^15^, and many studies have demonstrated that mercury anomalies are linked with large igneous provinces (LIPs) and mass extinctions^16–27^, although not all LIP eruptions perturbed the Hg cycle^28^. Using Hg enrichment data, Racki *et al*.^25^ demonstrated a volcanic trigger for the Frasnian–Famennian biotic crisis and suggested this was the Centre Hills volcanics in Central European successions. Mercury enrichments have also been described for the middle and latest Permian extinctions^18,21,26,27^. Sanei *et al.’s*^18^ study of the latest Permian mercury enrichment in the Canadian High Arctic, attributed this to emissions from the Siberian Traps with deleterious environmental consequences. Wang *et al*.^26^ showed high Hg/TOC spikes (up to 900 ppb/wt%, relative to a background of <100 ppb/wt%) coincided exactly with the latest Permian extinction horizon (South China). In another Hg-extinction link, Kwon *et al*.^27^ attributed elevated Hg/TOC levels from the middle-upper Permian Gohan Formation of South Korea to Emeishan LIP volcanism in South China. Elevated mercury concentrations are also known for younger mass extinction events such as the end-Triassic^22,24^ the end-Cretaceous mass extinctions^16,17,19,20,23^, as well as for the Palaeocene–Eocene Thermal Maximum^29^.

Until recently there has been no evidence for large-scale volcanism during the LOME and LIP making it one of the few Phanerozoic crises to not have the usual driver. This has changed with recent reports of Hg enrichments from South China and Laurentia at the time of LOME indicating volcanism may indeed have played a role in the crisis^30,31^. Here we extend the known record of Hg spikes in an Upper Ordovician to lowermost Silurian to a peri-Baltic, deep shelf succession from a borehole located in the Holy Cross Mountains (HCM, Poland). To examine the potential role of anoxia in Hg enrichment, we have compared Hg with other trace metals (e.g. Mo), pyrite framboid diameter analysis, and biomarker data.

## Materials and Methods

**Figure 1:**
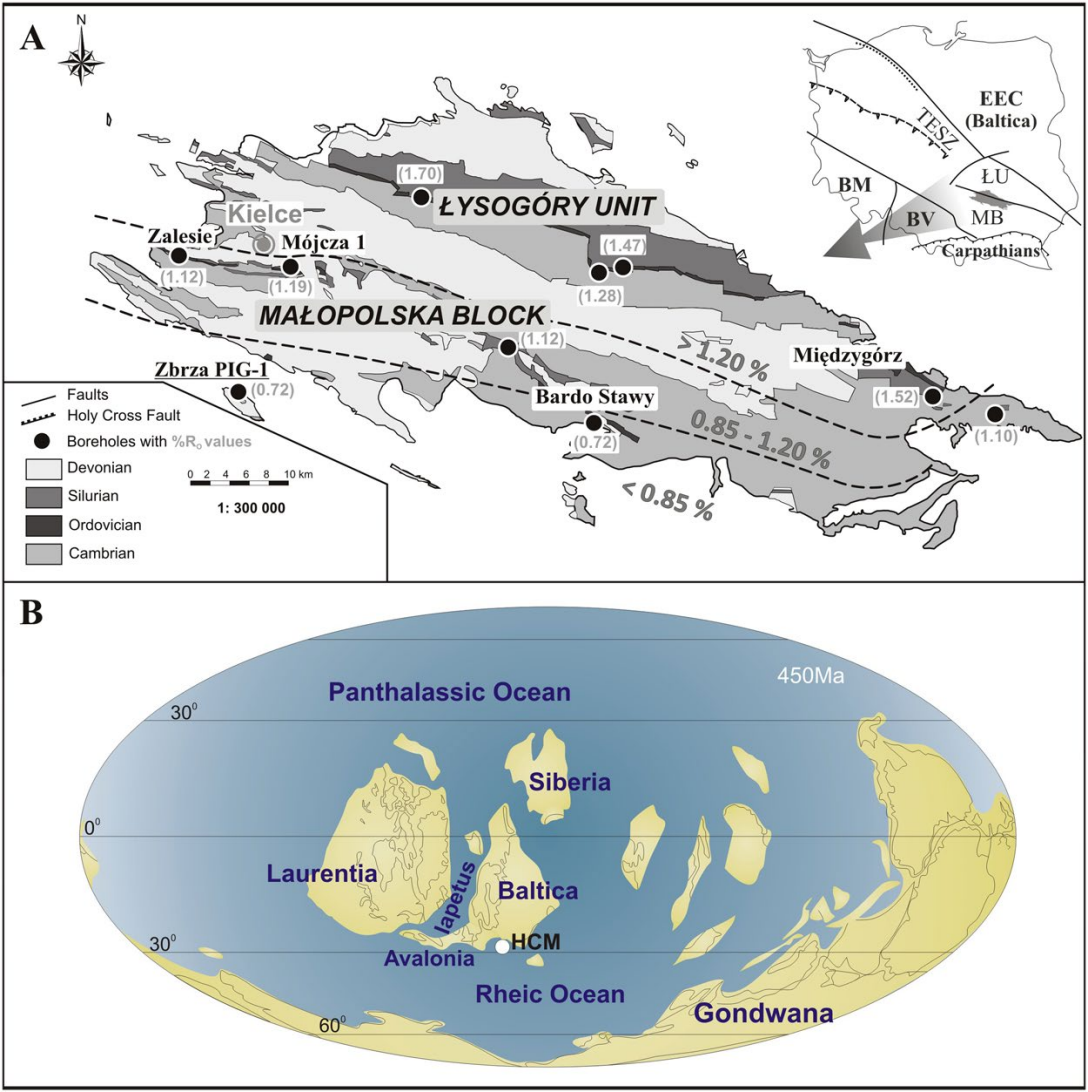
A. Simplified geological map of the Holy Cross Mountains showing the Zbrza PIG-1 location and maturation based on graptolite reflectance^33^. B. Map of Late Ordovician palaeogeography; HCM = Holy Cross Mountains. Inset in A: TESZ = Trans-European Suture Zone; BM = Bohemian Massif; BV = Brunovistulicum Massif; MB = Małopolska Block; ŁU = Łysogóry Unit; EEC = East European Craton.

**Figure 2:**
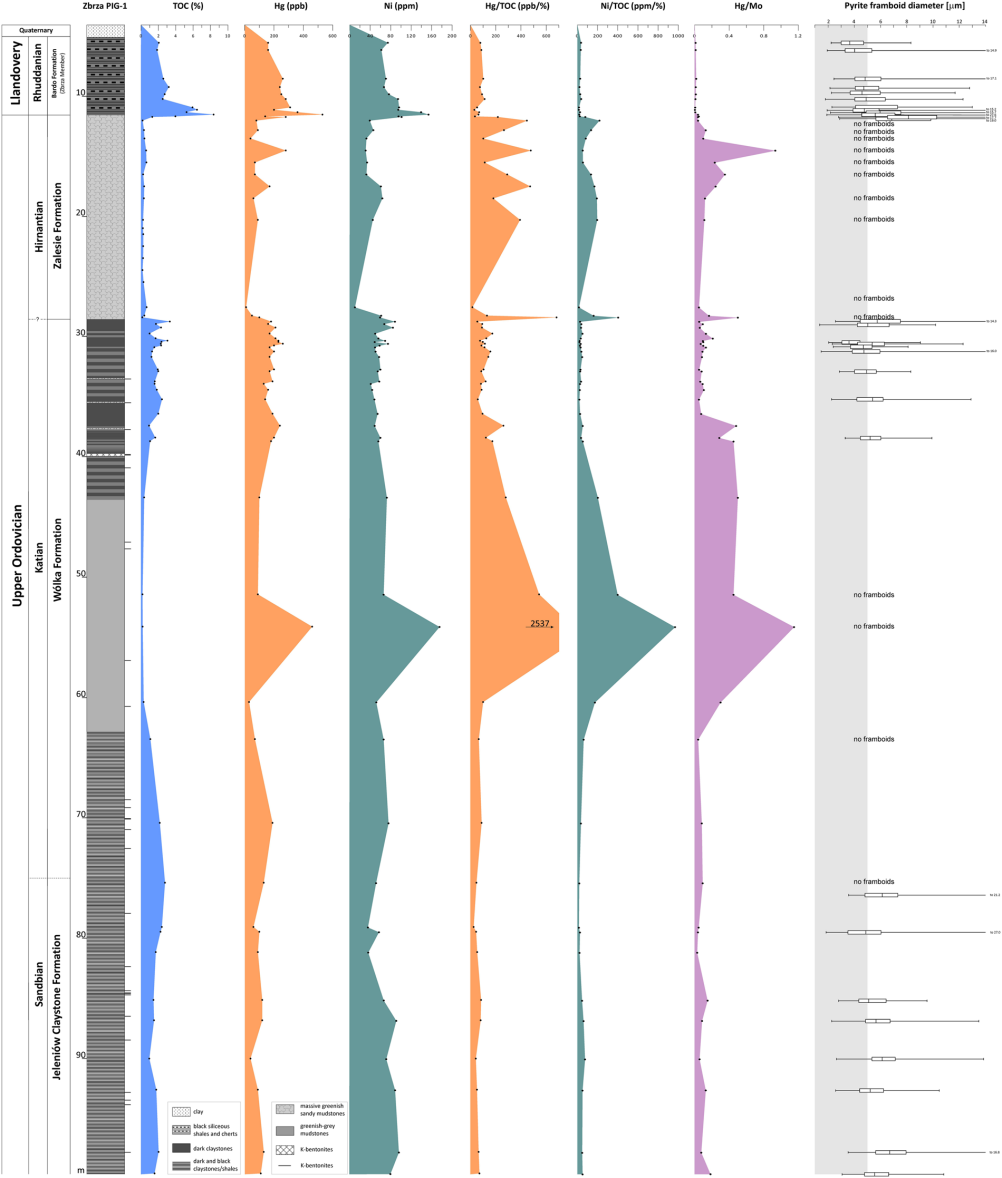
Stratigraphic variation of bulk geochemical data in the Zbrza section (see Table 1).

Sixty-eight samples were collected from the Zbrza PIG-1 borehole (Fig. 1), representing different types of Upper Ordovician and lower Llandovery claystone, mudstone, and black shale. The investigated samples are characterised by a relatively low level of thermal maturity (*ca* 0.72% of the vitrinite equivalent reflectance measured on graptolites)^32^. Upper Ordovician strata of the Zbrza PIG-1 borehole consist of the Sandbian to Hirnantian mudrock facies that form up to 90 m of the succession (Fig. 2). This shows laminae of varying thickness and is intercalated with numerous K-bentonite beds^33^. The Sandbian to lower Katian dark grey claystones and shales of the Jeleniów Formation are dated by their graptolite fauna that includes *Nemagraptus gracilis*, *Diplograptus multidens*, *Climacograptus wilsoni* and *Dicranograptus clingani*. They are overlain by the upper Katian clayey and marly mudstones and claystones of the Wólka Formation, which is roughly correlated with the *complanatus*, *complexus* and *pacificus* graptolite biozones. The mostly grey and greenish mudrocks of the Wólka Formation are intercalated, in their upper part, with dark grey and black shales (Fig. 2). Hirnantian strata in the Zbrza PIG-1 consists of sandy mudstones, marls and shales of the Zalesie Formation dated by *Mucronaspis* trilobites and the Hirnantia brachiopod fauna. There is a conspicuous facies change at the Ordovician/Silurian boundary with the Hirnantian mudstones overlain by Rhuddanian black, siliceous shales and cherts of the Zbrza Member. This is up to 20 m thick and is a component of the Bardo Formation. Graptolites indicate the *ascensus* to *cyphus* biozones are present and the Aeronian *triangulatus* biozone has also been postulated (Smolarek *et al*.^12^ *and references cited there*).

### Total organic carbon (TOC)

An ELTRA CS-500 IR-analyzer with a TIC module was used for total carbon (TC) and total inorganic carbon (TIC) determination. Total organic carbon was calculated as the difference between TC and TIC. For the calibration, means of the ELTRA standards were applied. There were better than ±2% for TC and ±3% for TIC analytical precision and accuracy. Analyses were performed at the Faculty of Earth Sciences, University of Silesia (Poland).

### Analysis of trace metals

Samples were analysed at Bureau Veritas AcmeLabs, Vancouver, Canada. For this study Ni and Mo concentrations of 20 samples were analysed, and the other trace element data comes from Smolarek *et al*.^12^. ICP-emission spectrometry following lithium borate fusion and dilute acid digestion of a 0.2 g sample powder was used for major oxides and several minor elements (Mo, Ni) measurements. The analytical results were compared with analyses of international standard reference materials and several samples were analysed in duplicate. Results, precision and accuracy were better than ±0.05% (mostly ±0.01%) for the major elements and generally better than ±1 ppm for the trace elements.

### Mercury determination

Two independent methods were used for mercury determination for 20 samples (in Table 1 marked by bold). Other Hg measurements were performed using inductively coupled plasma mass spectrometry. ICP-MS analysis was conducted commercially at AcmeLabs, Vancouver, Canada. The precision and accuracy of Hg determination were better than ±10 ppb.

**Table 1:** Concentrations and molecular parameters for the Zbrza PIG-1 borehole (nd – no data).

Sample	Stratigraphy	Lithology	TOC	CC	Hg	Mo	Ni	Hg/TOC	Hg/Mo	Ni/TOC	Me, *n*-Pr/Me,Et	ΣC13-C20
			%	%	ppb	ppm	ppm	ppb/%	ratio	ppm/%	ratio	ug/TOC
ZB 5.5	Rhuddanian	shales	2.0	0.0	160	13.8	74.4	78.3	0.01	36.4	0.041	0.31
ZB 6.1			1.9	0.0	160	13.2	61.7	86.4	0.01	33.3	0.030	0.06
ZB 8.5			2.6	0.0	260	13.8	70.7	101.4	0.02	27.6	0.039	0.27
ZB 9.2			3.2	0.0	240	18.2	66.9	75.2	0.01	21.0	0.039	0.40
ZB 9.8			2.7	0.0	250	17.9	76.6	91.8	0.01	28.1	0.044	0.27
ZB 10.2			2.5	0.0	280	28.8	94.4	112.2	0.01	37.8	0.023	0.06
ZB 10.9			5.9	0.0	310	47.3	96.4	52.3	0.01	16.3	0.047	0.15
ZB 11.1			6.4	0.0	200	53.0	94.9	31.1	0.00	14.7	0.043	0.38
ZB 11.3			5.2	0.0	360	46.9	139.4	68.7	0.01	26.6	0.043	10.33
*ZB 11.5*			8.4	0.0	530	13.6	153.9	63.4	0.04	18.4	0.047	0.28
ZB 11.65	Hirnantian	mudstones	4.0	0.0	140	2.9	95.3	35.4	0.05	24.1	0.064	0.22
*ZB 11.7*			1.3	0.0	280	7.2	101.3	216.8	0.04	78.4	0.090	0.08
ZB 12.0			0.2	21.4	80	2.0	39.2	446.7	0.04	218.9	nd	nd
*ZB 12.8*			0.3	13.8	90	0.7	46.1	265.2	0.13	135.8	0.0	0.00
ZB 13.5			0.4	34.1	40	0.4	32.6	101.8	0.10	83.0	0.0	0.00
ZB 14.5			0.6	52.2	280	0.3	31.1	477.5	0.93	53.0	0.0	0.00
ZB 15.5			0.6	65.2	70	0.3	33.9	113.2	0.23	54.8	nd	nd
*ZB 16.5*			0.2	39.5	70	0.2	32.5	291.0	0.35	135.1	nd	nd
ZB 17.5			0.4	39.2	170	0.7	60.8	472.5	0.24	169.0	0.0	0.60
ZB 18.5			0.3	25.8	60	0.5	63.7	181.8	0.12	193.0	nd	nd
*ZB 20.3*			0.2	7.0	90	0.8	45.0	392.3	0.11	196.2	0.0	0.92
ZB 21.0			0.2	17.9	nd	nd	nd	nd	nd	nd	0.0	0.0
ZB 21.5			0.3	21.5	nd	nd	nd	nd	nd	nd	0.0	0.0
ZB 22.5			0.2	11.9	nd	nd	nd	nd	nd	nd	0.0	0.0
ZB 23.5			0.2	5.0	nd	nd	nd	nd	nd	nd	0.0	0.0
ZB 24.5			0.2	7.0	nd	nd	nd	nd	nd	nd	0.0	0.0
ZB 25.5			0.3	6.2	nd	nd	nd	nd	nd	nd	0.0	0.0
ZB 27.6			0.6	81.9	10	0.2	10.1	15.6	0.05	15.7	nd	nd
ZB 28.3			0.4	3.4	50	0.3	61.4	130.7	0.17	160.5	nd	nd
ZB 28.45			0.1	1.2	100	0.2	59.3	681.3	0.50	404.0	0.0	0.0
ZB 28.8	Katian	claystones	3.3	1.0	180	3.4	88.3	54.4	0.05	26.7	nd	nd
ZB 29.0			1.7	0.0	160	1.7	67.5	93.2	0.09	39.3	0.106	1.75
ZB 29.3			2.3	0.0	210	3.5	84.3	91.3	0.06	36.7	nd	nd
*ZB 29.8*			1.0	2.2	170	1.3	49.7	173.2	0.13	50.6	nd	nd
*ZB 30.2*			1.7	0.2	210	1.0	56.0	125.3	0.21	33.4	0.091	3.24
ZB 30.4			3.0	0.1	230	2.4	69.1	75.7	0.10	22.7	0.096	0.64
*ZB 30.5*			2.4	1.2	230	2.3	48.7	97.8	0.10	20.7	nd	nd
ZB 30.65			2.3	0.1	260	3.7	74.7	114.2	0.07	32.8	0.075	2.99
*ZB 30.8*			2.3	4.2	200	2.0	58.3	86.7	0.10	25.3	0.055	3.31
*ZB 30.95*			1.5	0.9	170	1.3	49.4	111.3	0.13	32.3	nd	nd
*ZB 31.3*			1.3	1.0	200	2.2	50.4	157.5	0.09	39.7	nd	nd
*ZB 31.75*			1.2	0.0	170	2.0	57.4	141.3	0.09	47.7	0.100	1.63
*ZB 32.8*			1.9	0.6	200	3.9	59.9	104.0	0.05	31.1	0.092	1.65
ZB 32.95			2.0	0.0	170	2.1	54.7	85.7	0.08	27.6	nd	nd
*ZB 33.8*			1.6	0.1	190	2.9	57.7	120.0	0.07	36.5	0.096	0.74
ZB 34.0			1.6	3.4	130	1.4	41.1	83.2	0.09	26.3	nd	nd
*ZB 34.5*			1.8	2.6	160	1.5	43.8	88.2	0.11	24.1	nd	nd
ZB 35.3			2.4	6.8	140	2.8	48.2	58.2	0.05	20.0	0.041	4.16
ZB 36.5			2.0	0.1	190	2.5	54.4	95.6	0.08	27.4	nd	nd
*ZB 37.5*			0.9	0.5	240	0.5	48.7	261.1	0.48	53.0	0.0	0.83
*ZB 38.5*			1.6	0.1	200	0.7	59.9	122.2	0.29	36.6	nd	nd
*ZB 38.8*			1.0	0.0	180	0.4	55.9	173.6	0.45	53.9	nd	nd
ZB 43.5			0.4	0.1	100	0.2	72.5	279.7	0.50	202.8	nd	nd
ZB 51.6			0.2	0.0	90	0.2	66.1	543.5	0.45	399.2	nd	nd
*ZB 54.3*			0.2	0.1	460	0.4	175.4	2537.3	1.15	967.5	0.0	0.0
ZB 60.6			0.3	1.1	30	0.1	51.9	100.2	0.30	173.3	nd	nd
ZB 63.7			1.1	10.6	70	1.7	66.2	65.4	0.04	61.8	nd	nd
*ZB 70.7*			2.2	0.9	190	2.3	75.7	88.1	0.08	35.1	nd	nd
ZB 75.7	Sanbian	claystones	2.8	6.8	130	1.4	51.4	47.2	0.09	18.7	nd	nd
ZB 79.4			2.4	23.4	60	1.3	35.5	25.1	0.05	14.9	nd	nd
ZB 79.8			2.2	6.2	100	2.6	57.0	44.7	0.04	25.5	nd	nd
ZB 81.5			1.7	12.7	90	3.0	36.1	53.1	0.03	21.3	nd	nd
ZB 85.5			1.4	2.9	120	0.8	66.4	83.9	0.15	46.4	nd	nd
ZB 87.2			1.5	5.6	120	1.4	90.9	80.4	0.09	60.9	nd	nd
ZB 90.4			0.9	28.5	40	0.7	71.3	42.2	0.06	75.3	nd	nd
ZB 93.0			1.8	10.0	90	0.7	88.6	51.4	0.13	50.6	nd	nd
ZB 98.2			2.0	4.2	130	1.7	96.0	64.4	0.08	47.5	nd	nd
ZB 100.0			1.6	10.3	110	0.6	79.4	70.8	0.18	51.1	nd	nd

For atomic absorption spectrometry (AAS), a pyrolyser-type Milestone DMA-80 Direct Mercury Analyzer was used, with a detection limit of 0.2 ppb. DMA analytical curves were prepared with dilution of a 1-mg L^‒1^ standard solution (Merck Darmstadt, Germany). Duplicate measurements were taken of each sample; analyses were repeated when the coefficient of variability of the samples exceeded 5%. The instrument was calibrated prior to the measurement using certified reference material INCT-OBTL-5 (tobacco leaves) with a Hg content of 20.9 ppm. The measured error did not exceed 2% (for more details see Racki *et al*.)^34^. Analyses were performed at the Faculty of Earth Sciences, University of Silesia (Poland).

The correlation coefficient between ICP-MS and AAS mercury data was very good (R^2^ = 0.83). In this study we present Hg data obtained by the ICP-MS method.

### Analysis of pyrite framboid diameter

Forty-four rock samples from the Zbrza PIG 1 borehole (Fig. 1) were selected for framboidal pyrite diameter assay. Small chips of all samples were polished, and examined for pyrite framboids in an uncoated state using back-scattered electron (BSE) imaging with the aid of Philips XL30 Environmental Scanning Electron Microscope (ESEM) at the Faculty of Earth Sciences (Sosnowiec, Poland). The ESEM internal measuring device was used for measurement of the framboid diameters (in µm). At least 100 framboids were measured in each sample when possible. The results were presented in the traditional form of box and whisker plots^35^.

### Organic matter extraction and separation

An extraction of crushed (to *ca* 100 mesh) samples was made in an accelerated Dionex ASE 350 solvent extractor with a mixture of dichloromethane (DCM)/methanol (5:1 v:v). The column chromatography was used for the extracts separation into aliphatic, aromatic, and polar fractions^36^. Firstly, silica gel was activated at 120 °C for 24 h, then cooled and poured into Pasteur pipettes. The fraction collection was made by three eluents, namely, *n*-pentane for the aliphatic fraction, *n*-pentane and DCM (7:3) for the aromatic fraction, and DCM and methanol (1:1) for the polar fraction.

### Maleimide separation and derivatisation

Separation of the polar fraction obtained via column chromatography was made on activated silica gel in Pasteur pipettes. A fraction eluent a DCM/acetone mixture (8:2) was used. To obtain maleimide tertiary-butyl-dimethylsilyl derivatives, derivatisation of maleimide fraction was performed with MTBSTFA (*N*-*tert*-butyldimethylsilyl-*N*-methyltrifluoroacetamide). Samples were derivatised with MTBSTFA dissolved in super-dehydrated DCM, heated at 50 °C for 1 h, and run directly after derivatisation (see also Grice *et al*.)^37^.

### Gas chromatography‒mass spectrometry (GC–MS)

GC‒MS analysis was conducted using an Agilent Technologies 7890 A gas chromatograph and an Agilent 5975 C Network mass spectrometer with a Triple-Axis detector at the Faculty of Earth Sciences, Sosnowiec, Poland. The carrier gas was helium (6.0 grade) used at a constant flow of 2.6 ml/min. Separation was performed on either of two differently fused silica capillary columns: J&W HP5-MS (60 m × 0.32 mm i.d., film thickness 0.25 µm) coated with a chemically bonded phase (5% phenyl, 95% methylsiloxane) and J&W DB35-MS (60 m × 0.25 mm i.d., film thickness 0.25 µm) coated with a chemically bonded phase (35% phenyl, 65% methylsiloxane. Mass spectra were recorded from *m/z* 45–550 (0–40 min) and *m/z* 50–700 (>40 min). The mass spectrometer was operated in the electron impact mode (ionisation energy 70 eV).

## Results

Mercury concentrations were obtained for selected samples of the entire Zbrza PIG-1 profile (Figs 1 and 2; Table 1). The Sandbian claystones were characterised by low to elevated values of Hg ranging from 40 to 190 ppb, with a mean value of 105 ppb. The Hg concentrations in the Katian ranged from 30 to a very high value of 460 ppb (the mean value was 187 ppb). In the Hirnantian mudstones, mercury concentrations were generally low (approximately 10 to 90 ppb) with a few spikes of ~100 to 280 ppb (Fig. 2, Table 1). The youngest part of the profile recorded high values of mercury ranging from 160 to the highest value of the entire profile, 530 ppb (Table 1).

**Figure 3:**
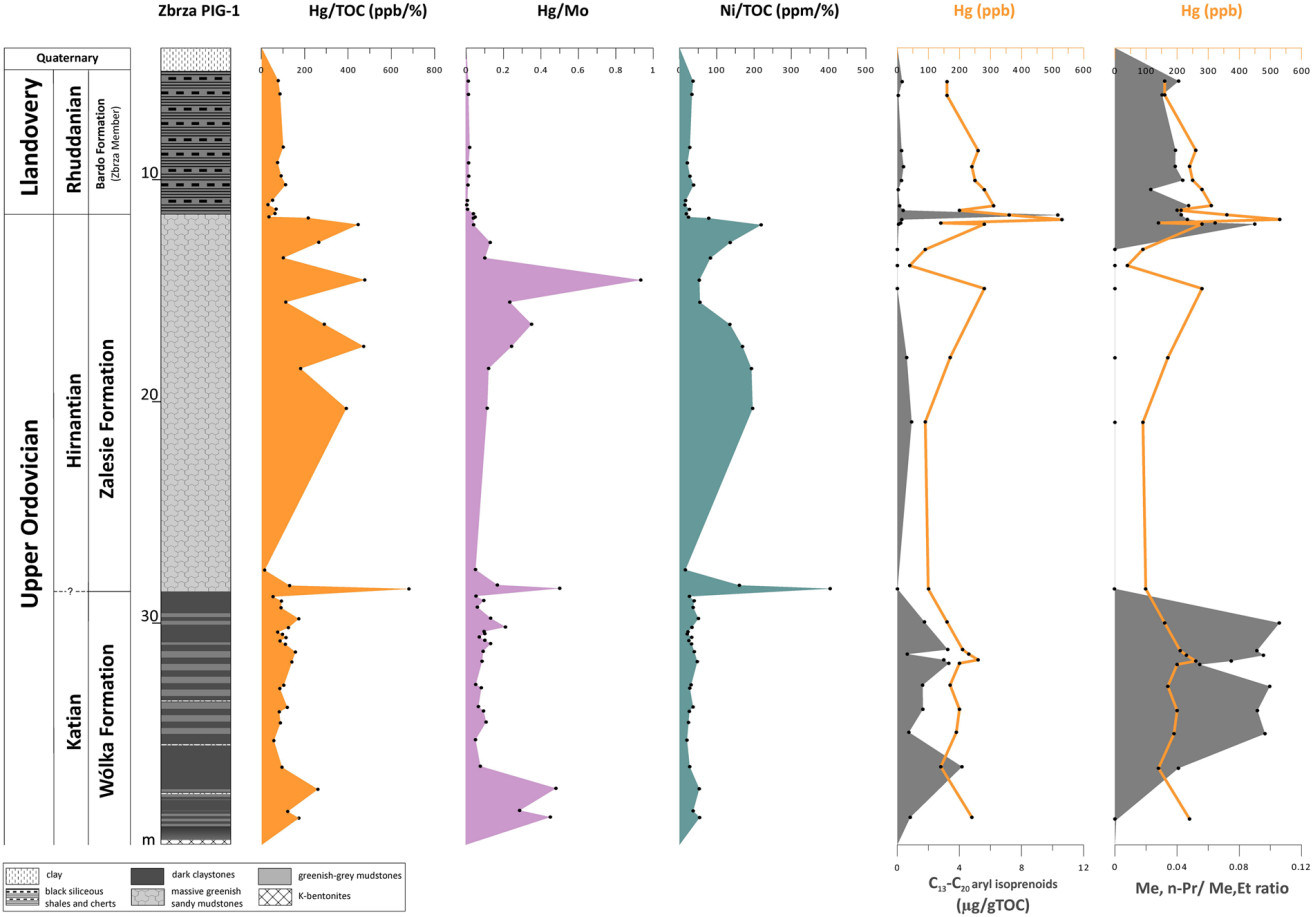
Stratigraphic distribution of trace metals and biomarkers for pre-LOME and LOME intervals (see Table 1; maleimide ratio ‒ Me, n-Pr/Me, Et).

Because Hg concentration and organic matter in sediments are closely correlated, the presentation of Hg results as a ratio of mercury to total organic carbon (Hg/TOC, ppb/wt%) is typically used. The Zbrza PIG-1 section is characterised by a low degree of correlation between Hg and TOC in the Sandbian (R^2^ = 0.2), with no positive Hg/TOC anomalies (Fig. 2, Table 1). No correlation exists between Hg and TOC in the Katian (R^2^ = 0.01), but strong positive anomalies were noted in the lower late Katian (Hg/TOC = 2537.3 ppb/wt%) and at the Katian-Hirnantian boundary (Hg/TOC = 681.3 ppb/wt%). No such correlation (R^2^ = 0.07) was distinguished in the Hirnantian, apart from several positive anomalies (Figs 2 and 3). The Silurian (Rhuddanian) shales and cherts showed a moderate correlation (R^2^ = 0.56) between Hg and TOC, with no positive anomalies (Figs 2 and 3). The Ni/TOC (ppm/wt%) ratio shows trends similar to those noted for Hg/TOC (Fig. 2, Table 1).

Additionally, in order to exclude an increase in Hg connected with anoxic conditions supporting TOC and Hg elevation, we calculated the ratio of Hg to Mo (Table 1, Figs 2 and 3). This ratio shows trends very similar to those of Hg/TOC values, with the same correlations and positive anomalies (Table 1, Figs 2 and 3).

In order to further check the possibility that Hg enrichment was caused by anoxic conditions, we assessed additional redox indicators. Thus, pyrite framboid diameters were measured for selected samples of the entire Zbrza PIG-1 profile (Fig. 2). Three intervals were found to display a large number of tiny (<5 μm) framboids, (in the Sandbian, the upper late Katian, and the Rhuddanian (Fig. 2). In contrast, no framboids were found in two intervals: the upper Sandbian to the lower late Katian and the entire Hirnantian (Fig. 2). Biomarkers of anoxic/euxinic conditions were also assessed for the Hirnantian and the O/S boundary intervals (Fig. 3, Table 1). The upper late Katian and the Rhuddanian, both characterised by elevated TOC values, contain both maleimides and aryl isoprenoids (Fig. 3, Table 1). The Hirnantian interval shows elevated mercury values, but euxinic biomarkers are absent or present in very low level of abundance (Fig. 3, Table 1).

## Discussion

### Mercury vs total organic carbon

Phanerozoic mass extinctions have been linked to the emplacement of LIPs^38–40^ and the associated release of SO_2_ and CO_2_^6,41,42^. Because mercury is the major pre-anthropogenic product of volcanism in Earth’s surface environments^43^, its accumulation in sediments is commonly used as a proxy for ancient volcanic events^18,22,25^. Changes in the content, early diagenetic degradation, or evaluated environmental loading of organic matter may influence both Hg and Hg/TOC enrichments^30^. In the case of the Zbrza section, in both the Sandbian and Rhuddanian stages, Hg was correlated with TOC content (R^2^ = 0.2 and 0.56, respectively), which does not indicate environmental Hg loading, especially in the case of Rhuddanian organic-rich shales. The opposite was true in intervals connected with LOME. No correlation was found between Hg and TOC content for either the late Katian (R^2^ = 0.01) or the Hirnantian (R^2^ = 0.07) (Fig. 2), suggesting that this pattern of Hg concentration was not a result of changing TOC content^30^. Moreover Jones *et al*.^30^ emphasised that the Hg/TOC ratio may be diagenetically inflated by loss of TOC during burial. In the Zbrza section all samples exhibit a rather high TOC value (specifically, ≥0.2 wt%), with the exception of sample ZB 28.45 (at the Katian-Hirnantian boundary), which has a low TOC content (0.1 wt%). However, due to the generally low level of thermal maturity of the Zbrza samples^33^, the loss of carbon during diagenesis is unlikely (Fig. 2, Table 1).

Moreover, we used Ni/TOC (ppm/wt%) to support the use of mercury as a volcanic indicator. Recently, Rampino *et al*.^44^ suggested a nickel anomaly as a link between the Siberian Traps eruptions and the latest Permian mass extinctions. For the Zbrza section, the Ni/TOC ratio corresponded to Hg/TOC data, with anomalies in the late Katian (Ni/TOC~967 ppm/wt%; Ni~175 ppm, while the background is approximately 65 ppm), on the Katian-Hirnantian boundary (Ni/TOC~404 ppm/wt%), and in the late Hirnantian immediately below the Ordovician-Silurian boundary (Figs 2 and 3; Table 1).

### Mercury’s independence of redox-sensitive indicators

The strongest evidence for the occurrence of euxinic (persistently sulfidic lower water column) conditions in the photic zone of the water column is the identification of biomarkers from green sulphur bacteria (GSB)^45,46^. The most powerful and frequently used indicators of euxinia are isorenieratane and its diagenetic degradation products^47–51^ and Me,i-Bu maleimides, which are degradation products of bacteriochlorophyll pigments related to autotrophic sulphur bacteria (*Chlorobiaceae*)^52–54^. Framboidal pyrite populations provide another redox-sensitive indicator^35^. Based on Wignall & Newton^35^, we interpreted euxinic conditions for the samples with high number of very low-diameter pyrites (mean 3–5 μm; narrow size range), anoxic conditions for the samples with high number of low-diameter pyrites (mean 4–6 μm; some larger framboids), samples where framboids were moderately common with broad range of sizes were interpreted as dysoxic conditions and oxic conditions for the samples lacking framboids. It is noteworthy that intervals with strong positive Hg/TOC anomalies (late Katian, Katian-Hirnantian boundary, late Hirnantian) are characterised by the absence of ‘anoxic indicators’ such as biomarkers (aryl isoprenoids, maleimides) or framboidal pyrite (Figs 2 and 3; Table 1). Thus, these Hg spikes appear to be unconnected with organic matter accumulation in anoxic/ euxinic conditions. Moreover, the Zbrza section displays the high Hg/Mo ratio. Mo, as a redox-sensitive element, is associated with pyrite^55^ and increases in euxinic conditions. The three highest Hg/Mo spikes occur in the lower late Katian, at the Katian-Hirnantian boundary, and in the late Hirnantian, showing Hg’s independence of Mo and other redox-sensitive elements. Gong *et al*.^31^ in their study of Ordovician-Silurian strata from South China, presented Hg and Mo concentrations. As was true of the Zbrza section, their Hg/Mo spikes were related to Hg/TOC anomalies, which they interpreted as Hg flux to the ocean basin.

To sum up, the lack of strong Hg/TOC correlation, in combination with high levels of TOC concentration, the presence of Ni enrichments, and the absence of ‘indicators of anoxia/euxinia’ (no specific biomarkers, no framboids, very low Mo concentration), supports the interpretation that the positive Hg/TOC anomalies in the lower late Katian, on the Katian-Hirnantian boundary, and in the late Hirnantian (Figs 2 and 3) record enhanced environmental Hg loading.

### Pre-LOME and LOME volcanic event

**Figure 4:**
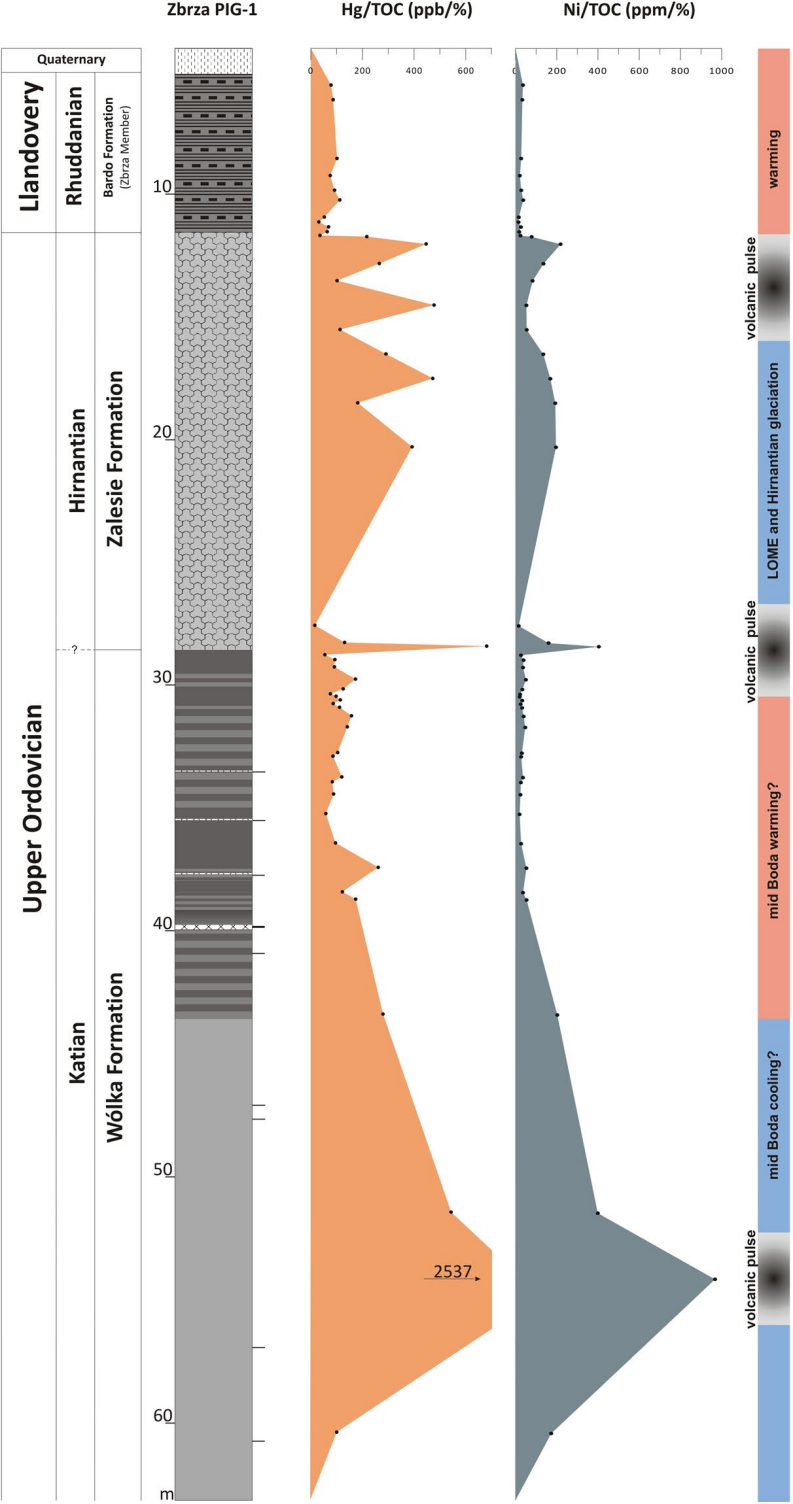
Stratigraphic distribution of mercury data for pre-LOME and LOME intervals of Zbrza PIG-1 with an interpretation of climate changes.

The Katian and Hirnantian strata in the Zbrza section display both Hg and Hg/TOC anomalies with a rather high concentration of TOC (≥0.2 wt%) at the same stratigraphic interval (Table 1, Fig. 2). For the lower late Katian, Hg reached approximately 3 times the background level (Hg/TOC ~2500 ppb/wt%) and can be roughly correlated with the mid-Boda change from cool to warm climate conditions^4^. The spike detected precisely on the Katian-Hirnantian boundary shows high values of Hg/TOC (~681 ppb/wt%), as well as a positive anomaly of Ni/TOC (~404 ppm/wt%). The late-Hirnantian anomaly is approximately double the Hg background level (Hg/TOC~470 ppb/wt%) and appears to be coeval with the termination of the Hirnantian glaciation (Table 1, Figs 2 and 4). Similar positive Hg/TOC anomalies were also reported in Wangjiawan (South China) and the Monitor Range (West Laurentia) by Jones *et al*.^30^, who distinguished the *ornatus* (=*complexus*) anomaly in the late Katian, coincident roughly with the lower late Katian anomaly from Zbrza, but characterised by a lower concentration of Hg and moderate TOC content (0.10–0.19%). The Katian-Hirnantian anomaly from the Zbrza section can be compared to the first pulse of the *pacificus* anomaly^30^ with extreme Hg enrichment (500 times background levels). Jones *et al*.^30^ postulate that Hg enrichments are products of enhanced environmental loading driven by LIP emplacement. Thus, the Hg enrichment in the Katian geochemical record (the *ornatus* anomaly) is interpreted as a volcanic event, that triggered severe cooling^30,56,57^. It has been suggested that the upper *pacificus* anomaly is connected with a volcanic eruption which triggered an albedo catastrophe and the rapid expansion of ice sheets^30^. The presence of numerous K-bentonites in the Upper Ordovician of the Zbrza section suggests that the Hg spikes may be related to increased delivery of volcanic ash. Trela *et al*.^32^ postulate that pyroclastic material was transported to the HCM by westerlies from the Avalonian volcanoes. Alternatively, Shen *et al*.^58^ claimed that expanded eukaryotic algal production contributed to a higher level of export efficiency during the late Katian, resulting in increased organic carbon burial and drawing down of CO_2_ which ultimately triggered the Hirnantian glaciation. Enhanced algal production may have been connected with the delivery of nutrients to the ocean from volcanic sources. In the Zbrza section, the late-Hirnantian Hg anomaly was probably connected with the second phase of LOME, which can be linked with the global environmental effects of LIP-associated CO_2_ (marine transgression, warming, anoxia)^4,42^. Jones *et al*.^30^ argued that the late-Hirnantian warming was caused by CO_2_ release during the later phase of LIP emplacement and reduction of SO_2_ emissions^59^ as aerosol albedo forcing waned.

## Conclusions

We have identified three mercury spikes from the Katian and Hirnantian mudstone facies in a deep shelf area of the peri-Baltic area (Holy Cross Mountains, Poland) which we link to volcanic pulses that triggered massive climate changes during the Late Ordovician mass extinction. A comparison of Hg concentration with biomarker, pyrite framboid, and molybdenum data showed that the intervals of organic matter enrichment are associated with anoxia, but are not related to the late-Katian and late-Hirnantian Hg anomalies. As in the case of other mass extinctions, we recorded Hg/TOC anomalies immediately below the interval of the second pulse of the Ordovician extinction. Two volcanic pulses are directly related to warm periods during the Ordovician and Silurian, while the third, precisely on the Katian-Hirnantian boundary, corresponds to Late Ordovician cooling (glaciation).

Although no late-Katian‒Hirnantian LIPs have been identified to date, we conclude that our Hg and Hg/TOC anomalies in the late Katian and Hirnantian were associated with volcanic pulses which triggered major environmental changes and, consequently, the Late Ordovician mass extinction. Since there is no direct geological or geochronological evidence for late-Katian‒Hirnantian LIPs, more studies are required. Identification of a Late Ordovician LIP would indicate that the LOME was due to a volcanism-driven climate changes mechanism.

## Data Availability

All data generated or analysed during this study are included in this published article. All figures were created by authors of the paper.
